# Contamination Characterization, Toxicological Properties, and Health Risk Assessment of Bisphenols in Multiple Media: Current Research Status and Future Perspectives

**DOI:** 10.3390/toxics13020109

**Published:** 2025-01-29

**Authors:** Fangyun Long, Yanqin Ren, Fang Bi, Zhenhai Wu, Haijie Zhang, Junling Li, Rui Gao, Zhengyang Liu, Hong Li

**Affiliations:** State Key Laboratory of Environmental Criteria and Risk Assessment, Chinese Research Academy of Environmental Sciences, Beijing 100012, China

**Keywords:** bisphenols (BPs), detection method, potential hazards, physical properties, health assessment

## Abstract

Bisphenols (BPs) are ubiquitous environmental endocrine disruptors that cause various human health hazards and pollute water, soil, and the atmosphere to varying degrees. Although various studies have investigated the pollution characteristics and health hazards of BPs in different media, a systematic review of BPs in the broader environmental context is still lacking. This study highlights the pollution characteristics, detection methods, and risk assessment status of BPs by combining relevant studies from both domestic and international sources, and their environmental distribution characteristics are summarized. The results show that BP pollution is a widespread and complex global phenomenon. Bisphenol A (BPA) remains the predominant component of BPs, which can damage the nervous and reproductive systems. At present, high-performance liquid chromatography–tandem mass spectrometry, high-performance liquid chromatography, and liquid chromatography–tandem mass spectrometry are the main detection methods used for BPs. BPs can also damage the reproductive system, leading to germ cell apoptosis and ovarian damage. Future research should focus on expanding the BP testing repertoire, advancing rapid detection techniques, elucidating toxic mechanisms, conducting comprehensive safety assessments, and developing systematic health risk assessment methods. These efforts will provide a scientific foundation for preventing and controlling emerging pollutants.

## 1. Introduction

Emerging contaminants (ECs), which are a class of natural and synthetic chemicals and biological agents, have recently become a major focus of international environmental attention [1]. As ECs, endocrine-disrupting chemicals (EDCs) have gained increasing public attention and are now a topic of global concern regulated by international conventions. EDCs are defined as exogenous substances that cause adverse health effects in an intact organism [2]. EDCs have been reported to affect endocrine system functions, interfering with hormone action [3]. They have various sources, being commonly found in agricultural supplies, plastics, food contact materials, and personal care products. They have the potential to infiltrate the human body via various routes and affect the endocrine, reproductive, and nervous systems [4,5,6,7]. Bisphenols (BPs) are an important group of chemical raw materials commonly used to synthesize polymer materials, including bisphenol A (BPA) and its alternatives. These materials show favourable optical properties and exceptional high-temperature stability [8]. Meanwhile, BPs act as estrogen-like endocrine disruptors and potentially affect human health [9,10]. BPs can affect the reproductive neuroendocrine system via estrogen receptors and the aromatase pathway [10]. Further studies have shown that the immune system is particularly sensitive to BPA, especially mouse Th17 cells, which play a key role in cellular immune mechanisms [11]. Meanwhile, another study also found that some BPs can have a certain impact on the innate immune response; for example, BPA and bisphenol AF (BPAF) can reduce the transcriptional activity of RACK1 promoters, mRNA expression, and protein levels [12]. Today, dendritic cells are recognized at the center stage of adaptive, as well as innate, immune responses and are known to be the most important antigen-presenting cells [9]. Recently, experimental studies have shown that BPA and BPAF can significantly reduce the endocytotic capacity of human monocyte-derived dendritic cells (MDDCs) [9]. This also suggests that exposure to BPs may increase the risk of adaptive and innate immune responses in humans. In addition, BPs, as a class of endocrine disruptors, can further affect the regulation of other pathways, thus triggering tumor transformation [13]. Therefore, it is imperative to conduct more in-depth studies on the effects of BP exposure. BPA is one of the most representative BPs and typical EDCs. It was first synthesized in 1891 [5]. To date, it has been detected in various environmental media, including air, water, soil, dust, and sediment. According to statistical data, the annual production of BPA consistently increased by 4.6% from 2013 to 2019 [5,14,15,16,17]. Toxicological studies have demonstrated that BPA exerts considerable endocrine-disrupting effects, impacting human reproductive and nervous systems. Furthermore, evidence suggests that BPA exposure may be associated with autism spectrum disorder (ASD), polycystic ovary syndrome (PCOS), and other health conditions [18,19]. The use of BPA in packaging compounds for bottles, straws, and formulas has been prohibited in China, Canada, the European Union, and the United States (US) by the Food and Drug Administration (FDA) due to its multiple related health risks. These bans were implemented in 2011 (China), 2008 (Canada), 2011 (the European Union), and 2013 (the US). Moreover, France has also prohibited the use of BPA in any food or beverage packaging, with the ban taking effect in 2015 [16,20,21,22]. Studies have focused on finding alternatives to BPA due to increasing consumer demand and regulatory changes. Reports indicate that studies on BPA alternatives and their analogues have increased annually from 2000 to 2020 [23]. In January 2014, the US Environmental Protection Agency (EPA) Design for the Environment Program assessed chemical alternatives. The assessment included the potential impacts of BPA alternatives in thermal paper on human health and the environment [24]. BPA and its replacements may have multiple sources. For example, studies have discovered that BPA is commonly found in the polycarbonate plastics and epoxy resin industries. It is chemically bonded to polymers and difficult to disperse directly into the air. Therefore, solid waste incineration is one of the most significant sources of BPA in the environment [25]. Other bisphenol compounds are widely used as alternative compounds for BPA due to their similar structure, including bisphenol S (BPS), BPAF, bisphenol F (BPF), and bisphenol Z (BPZ) [8]. BPS has enhanced thermal and optical stability relative to BPA. It is predominantly used as an anti-corrosion agent in epoxy adhesives, a reagent in polymer reactions, and a developer [8,23]. BPAF is used as a crosslinker for fluoropolymers and as a monomer for other polymers, such as polyimides, polycarbonates, and polymers used in food contact applications [26]. Among the BPA alternatives, BPFA and BPS have the highest detection rates in human and environmental samples [27]. BPF is used in the production of thermal paper, in coating materials, and in the plastic processing industry [21]. BPZ is commonly used in the production of cured high-heat-resistant plastic materials [28]. Currently, BPs are widely used in various production and living applications [16]. However, there is a lack of global research investigating the presence of BPs in the environment. Moreover, BPs have a natural resistance to biodegradation, which poses a challenge for future treatment [8]. Thus, it is necessary to systematically summarize and address the previous research on BPs.

This study elucidates the transfer and pollution characteristics of BPs in three main environmental media (water, soil, and the atmosphere) via literature research and analyses. It also preliminarily investigates the transfer mode of BPs in these environments, providing a specific theoretical foundation for further study of BP pollution in the environment. A total of 21 BPs are assessed in terms of their physical properties, and their distribution coefficients are calculated using EPI software https://www.cdc.gov/epiinfo/index.html (accessed on 9 January 2025). The distribution in each phase is further examined. This study serves as a contribution to future targeted detection efforts. Furthermore, the main BP detection methods are summarized, the similarities and differences between the various methods are compared, and the potential risks of BPs in different media are roughly evaluated. The current prevention and control strategies for BPs are also summarized, providing technical and policy support for future BP pollution management efforts. This study provides a scientific basis for future research on the detection and health risk assessment of BPs, and it contributes to the development of scientific approaches for addressing emerging pollutants.

## 2. Pollution Characteristics

### 2.1. Physical Properties

**Figure 1:**
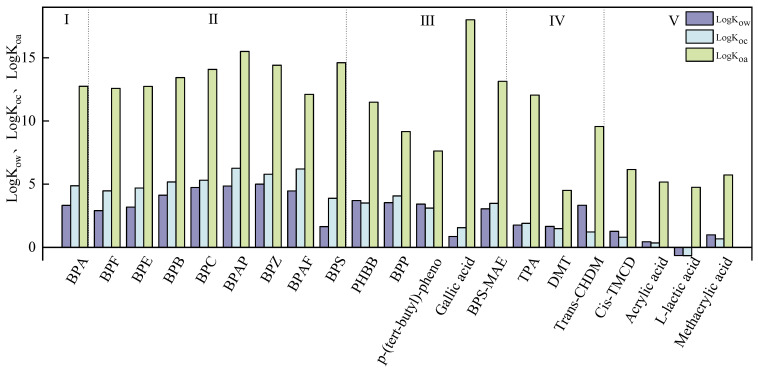
EPI simulation of each BP distribution coefficient (I: BPA; II: BPA analogues; III: BPA replacement compounds with one phenol; IV: BPA replacement compounds with aromatic rings; V: acyclic compounds). EPI Suite^TM^ provided by the EPA was used to calculate several parameters of BPA and its substitutes. The log octanol-water partition coefficient, log K_ow_, of chemicals is estimated using an atom/fragment contribution method; the Henry’s Law constant (air/water partition coefficient) is calculated using both the group contribution and the bond contribution methods; and K_oc_ is estimated using two different models: the Sabljic molecular connectivity method with improved correction factors and the traditional method based on log K_ow_. After EPI software calculates logK_ow_, logK_oa_, and logK_oc_ parameters of each substance, origin software is used to plot the data.

In industrial production and application, the selection of chemicals based on analogous physical properties is paralleled by considering alternatives with structurally related compounds [29]. Thus, performing a comparative evaluation between the original chemical and its alternatives is crucial to ensure that the substitutes do not induce the same or more pronounced biological effects while maintaining optimal physical characteristics. This approach aims to reduce the potential risks associated with chemical hazards. BPA alternatives frequently show properties analogous to those of BPA or more superior structural characteristics [8,21,23,28]. To date, approximately 16 bisphenol analogues have been well documented for industrial applications [8]. Based on their structural characteristics, BPA alternatives can be broadly classified into five categories: BPA analogues, compounds with a single phenol group, compounds with aromatic rings, compounds with non-aromatic rings, and acyclic compounds [23]. Among the five categories of BPs, BPA analogues are the most prevalent. They are mostly used to replace BPA as a monomer in epoxy resins and polycarbonate plastics [5]. In this study, 21 types of BPs, including 8 types of BPA analogues, 5 types of monophenol-containing compounds, 3 types of aromatic ring-containing compounds, 3 types of non-aromatic ring compounds, and 3 types of acyclic compounds, were examined. All physical parameters are shown in Table S1. Most BP substances have high melting and boiling points and are insoluble in water. The physical and structural properties of BPA and its replacements are comparable, but some BPA alternatives show distinct physical features from BPA. The water solubility of BPZ is considerably lower than that of BPA (172.7 mg/L), being only 1.5 mg/L. In contrast, gallic acid has a solubility of 1.2 g/L. The boiling point of L-lactic acid is only 122 °C, while BPA has a higher boiling point of 364 °C, which is triple that of L-lactic acid. Newly developed BPA alternatives may differ from BPs and may have different effects. Therefore, it is necessary to pay attention to the physical properties of BPA substitutes. In this study, EPI Suite^TM^ provided by the EPA, Washington, D.C, US was used to calculate several parameters of BPA and its substitutes. EPI is a Windows-based suite of physical/chemical property and environmental fate estimation programmes developed by the EPA and Syracuse Research Corp (SRC), New York, US [30]. EPI was used to calculate the following three parameters of the 21 BPs: the octanol–water partition coefficient (K_ow_), the soil–water partition coefficient (K_oc_), and the air–water partition coefficient (K_oa_). The different distribution coefficients can preliminarily indicate the distribution tendency of each substance in the environmental media [5]. The calculation results are shown in Table S2 and Figure 1. It can be observed that BPA and BPA analogues have a higher LogK_ao_, LogK_ow_, and LogK_oc_, and LogK_ao_ is much higher than LogK_ow_ and LogK_oc_. Most of the BP substances show a high LogK_oa_, which suggests that they are primarily distributed in the air. This is consistent with the low water solubility of BPs in terms of the above physical properties. It can be seen that the proportion of BPA and BPA analogues with a high LogK_oa_ is larger, while the LogK_ao_ of acyclic compounds is relatively small. Compared with BPA and BPA analogues, compounds with one phenol and aromatic ring, acyclic compounds tend to be more hydrophilic. Based on these results, the BP content in the three media is ranked in the order of air > soil > water, indicating that the identification and study of BPs in the atmosphere and soil environment may be enhanced in the future.

### 2.2. Environmental Transmission

**Figure 2:**
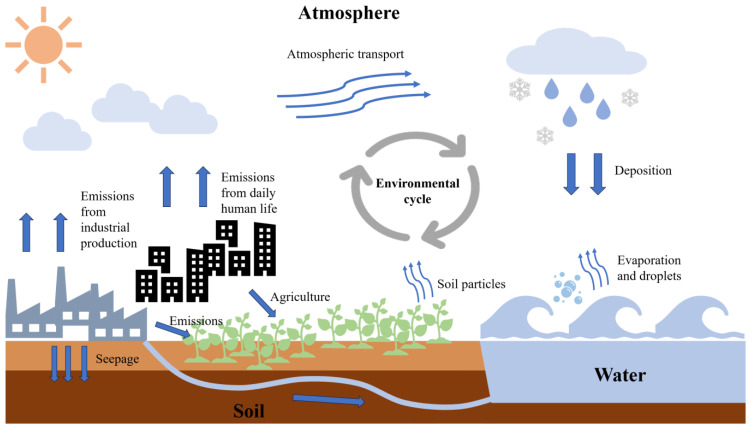
Schematic diagram of BP propagation in various media [32,35,36,37,38]. Industrial production, daily life, and agricultural production release BPs to the atmosphere, soil, and surface water. The BPs in the atmosphere diffuse through long-distance atmospheric transport and return to the soil and water through dry and wet settlement, the BPs in the soil surface penetrate into the groundwater and the groundwater flows into the surface water, and the BPs in surface water enter the atmosphere and soil through evaporation and contact so as to achieve environmental circulation.

In daily life, BPA is mainly used as a plasticizer, and it can be detected in specific medical devices and plastic products, including baby bottles and plastic bags [31]. Factors related to human activities, such as population density, economic development, and sewage discharge, will inevitably have an impact on the BPA content in the environment [32]. However, due to the restrictions placed on BPA by many regulations, its use is decreasing. To meet the needs of BPA use in daily life, BPA alternatives were developed, and these are expected to progressively infiltrate the environment through the production process and daily life activities [8,21,23,28,33]. Pollutants released into the environment can be dispersed through different mechanisms, such as atmospheric transmission [34], bubble rupture [35], infiltration [36,37], and dry and wet deposition [38] across interconnected water, soil, and atmospheric media. These processes contribute to the broader dissemination of pollutants, thereby expanding their diffusion range. Consequently, it is imperative to further investigate BPs’ mode of transmission in order to better understand the pollutant’s impact on the environment. As demonstrated by regional studies, BPA is found in atmospheric particles of coarse and fine sizes [34]. Soil resuspension is a source of BPA in atmospheric coarse particles; BPA is attracted to these particles, which eventually permeate the atmosphere. A previous study found that a large number of bubbles were generated when waves broke on the ocean, and these bubbles eventually burst and ejected water droplets, which played a key role in the formation of aerosols in the atmosphere [35]. This role also provided a basic framework for the transfer of substances at the water–gas interface [35]. Moreover, this framework provides a basis for the transfer of BPA from water to the atmosphere. However, relevant research data on BPA in marine aerosols are still limited. In the future, the transfer of BPA in sea air can be further studied. Furthermore, a survey found that a flat terrain slowed down the horizontal runoff rate and surface diffuse infiltration caused by atmospheric precipitation and agricultural irrigation; it also found that the vertical and lateral seepage of surface water polluted groundwater with BPA [36]. The infiltration of reclaimed water also polluted underground aquifers [37]. Another study analyzed roof-collected rainwater and found BPA, confirming that atmospheric deposition contributes to BPA pollution by returning it from the atmosphere to soil and surface water [38]. These results indicate that BPA can be transmitted in the environment through interactions at environmental interfaces and various physical mechanisms (Figure 2).

**Figure 3:**
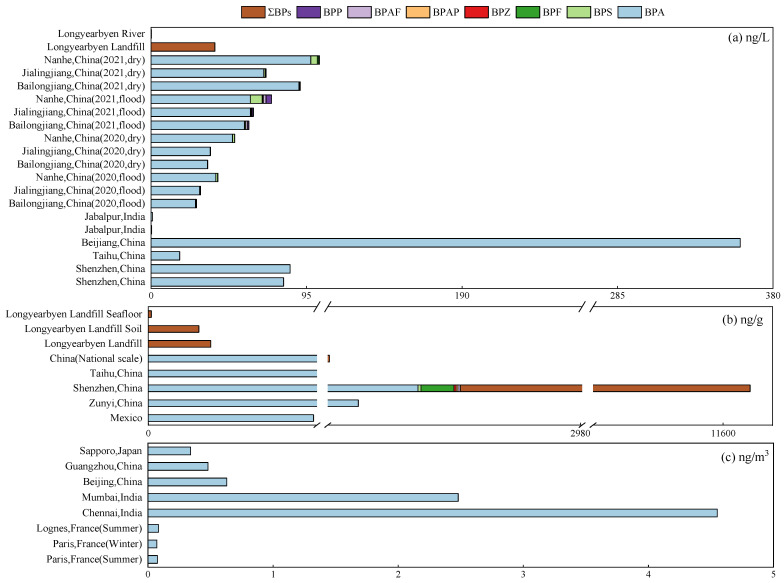
Concentration distribution of BPs in three environmental media: water, soil, and the atmosphere ((**a**) water; (**b**) soil; (**c**) atmosphere) [5,15,22,34,39,41,42,44,45,46,47,48,49,51]. China’s National Knowledge Infrastructure (CNKI) database was selected as the core data source. In CNKI, BPs, soil, atmosphere, and water are used as keywords for search. The detection data are selected in different environments at home and abroad, and Origin software is used for analysis and mapping.

Most recent test results show that, in the environment, the concentration of BPA is higher than that of other BPs. However, the detection of BP concentration has recently become very limited, particularly in the atmosphere. This review summarized the concentrations of BPA and six BPA analogues in three environments, namely, water, soil, and the atmosphere, as shown in Figure 3 and Table S3.

Human activities and daily necessities are key drivers of water pollution, leading to an increase in BP concentrations in natural water systems due to the discharge of urban domestic wastewater. Moreover, factors such as reduced rainfall, decreased river runoff, a lowered water self-purification potential, and impaired pollutant migration and transformation during the dry season collectively contribute to the accumulation and increased concentration of pollutants in water [39]. The number of sewage outlets and the use of industrial BPs also affect the pollution level of ΣBPs in water. Currently, the concentration of BPA in the global water environment ranges from about 0.09 × 10^−3^ to 228.04 µg/L [40], being much higher than that of other BPs. For example, in 2021, the Jialing River had the highest concentration of BPA in the wet season at 168.57 ng/L, while the highest concentrations of BPS, BPZ, BPAP, BPAF, and BPP were 1.44 ng/L, 5.7 ng/L, 1.36 ng/L, 3.3 ng/L, and 4.6 ng/L, respectively [39]. Therefore, among the BPs, BPA is still the main pollutant. Several studies have also found seasonal differences in the BPA concentration in water, with the most obvious difference being between winter and summer, showing high seasonal characteristics in summer and low seasonal characteristics in winter [5,22,39,41,42]. A possible reason for this is that BPA is primarily leached in high-temperature, acidic, or alkaline environments [43]. Therefore, seasonal fluctuations in BPA may be related to changes in local water quality, including local water temperature, acidity, and alkalinity. BP pollution is also influenced by waste disposal. A previous study found ΣBPs at 39 ng/L in the leachate of the Longyearbyen landfill site, which was almost 140 times the concentration of ΣBPs in the Longyearbyen River detected during the same period [5]. Therefore, this part of the leachate may enter groundwater and surface water via infiltration and surface runoff. Further, the BPA contamination of food and drinks may result from direct contact with plastic products, and BPA concentrations may marginally increase under heating conditions [44].

BPs can enter soil through direct wastewater discharge, urban runoff, solid waste treatment, etc. Moreover, agricultural activities are also one of the sources of soil BPs [40]. BPA is commonly adsorbed on soil particles due to its low vapour pressure and large octanol–water partition constant [40]. The highest concentration of ΣBPs detected in soils in Zhejiang, China, is 382.5 ng/g [45], which is much higher than the concentration of ΣBP (2.0 ng/g) in soils in Longyearbyen [5]. The results of Mexican soil sample tests indicated that BPA is a major contributor to BPs [46]. In a comprehensive national survey of soil BP contamination in China, BPA was detected in 85% of samples, making it the most prevalent BP [47]. A 2017 soil study in Shenzhen, China, reported that the maximum concentration of BPA was 6783 ng/g, which was much higher than that of other BPs: BPS (333 ng/g), BPF (4913 ng/g), BPZ (225 ng/g), BPAP (26.9 ng/g), BPAF (331 ng/g), and BPP (<5 ng/g) [46]. However, a survey in Zhejiang Province, China, showed that BPs in soil accounted for the main pollution contribution of BPP [48]. This variation in the primary soil components of the local area may be due to differences in industrial discharge conditions and farming methods. Furthermore, BPs are also concentrated in sediments, and their adsorption and desorption by sediments also impact their migration and transformation. The content of BPs in mud bottom varies across basins and regions, and the concentration of ΣBPs in landfill sites is relatively higher than that in other regions [5,8,25,32,49]. Recent studies on the concentration of BPs in soil have been relatively limited. However, survey findings indicate that the concentration of BPs is still detected, even in the Arctic region, where background concentrations are comparatively low. Combined with the above K_ow_ calculation results, these findings underscore the urgent need to focus on BP contamination in soil.

The widespread application of BPs in various products results in their inevitable release into the atmosphere through human activities. These BPs may persist in the atmosphere due to interactions and mutual transmission between different environmental media. However, current research on other BPs in atmospheric environments is particularly limited, with the majority of studies concentrating mainly on the detection of BPA. Due to the strong bonding between BPA and polymers, BPA is not easily released directly from these materials. Thus, the primary source of atmospheric BPA is solid waste combustion. A study showed that BPA in indoor and outdoor air was less affected by temperature fluctuations, while BPA concentration levels were relatively consistent in the cold and warm seasons [48]. Indoor concentrations of BPA may be affected by floor type and furniture [50], which may lead to higher concentrations in indoor air; additionally, the content of BPA differs among indoor environments. In Shanghai, the highest concentration of BPA detected in an office setting was 2.77 µg/g, while in student dormitories, BPA levels were found to be as high as 4.7 µg/g [51]. The BPA concentration found in student dormitories was nearly twice that found in office environments, suggesting that interior decoration may be a source of indoor BPA. Differences in BPA concentration across different indoor settings highlight the influence of specific sources. It has been suggested that paper supplies, including thermal paper and other office supplies, could potentially be a source of indoor BPA due to the presence of BPA components [29]. As per the environmental distribution of BPs, it is predicted that BPs may be distributed in the air in large quantities. Thus, it is imperative to enhance the atmospheric detection of BPs and broaden the application of detected substances in the atmosphere to collect fundamental data for future studies of BPs in the atmosphere.

Based on the above discussion, BPs, as widely used additives, are detected in water, soil, and the atmosphere in various regions, producing extensive global pollution. Environmental conditions (e.g., local water quality acidity), daily requirements (e.g., decoration furniture), and human activities (e.g., agricultural activities and industrial emissions) are likely to have some effect on BPs in the environment. The in-depth development of multimedia BP pollution detection methods is the primary key to solving the problem of BP pollution. It is necessary to establish a more complete BP detection list, expand the detection range and content of BPs, and establish BP standard benchmarks in different environments and production conditions.

## 3. Analytical Methods and Detection Techniques

BPs are extensively distributed, being found in various aspects of human daily life, the natural surroundings, and even human waste. To better detect the content of BPs in the environment and human body and to determine their distribution, various efficient and convenient detection methods must be urgently adopted. Table 1 summarizes the commonly used detection methods for BPs and the new detection methods developed for BPA to provide theoretical and technical support for the detection of BPA alternatives.

To date, BPA detection technology has been continuously improved; however, conventional methods, including HPLC, liquid chromatography–tandem mass spectrometry (LC-MS/MS), UPLC-MS/MS, and gas chromatography–mass spectrometry (GC-MS), continue to be used [52]. The detection scheme is optimized by adjusting the pre-treatment methods and instrument settings. For example, acetonitrile/0.1% formic acid aqueous solution was used for gradient elution, the optimal elution volume (7 mL) was extracted thrice, and the BPA content in vegetable oil was determined using HPLC. This method had good linearity, a recovery rate of 90.8%, and a low detection cost [53]. The detection method based on UPLC-MS/MS can optimize the mass spectrum, air enrichment conditions, and elution solvent. A CSH C18 column and an 8:2 (*v*:*v*; 5 mL) methanol–water mixture were used for ultrasonic elution, and the recovery rate of this detection method was 68–119% [54]. Zhang et al. [55] used ZnCl_2_ as a catalyst to synthesize amino-functionalized poly (N-vinylpyrrolidone divinylbenzene) [P(NVP-DVB)]. They then used the prepared P(NVP-DVB) as an adsorbent to detect BPA in the aqueous environment using HPLC and solid-phase extraction. The recovery rate was 99.7%. This method is effective in both simulated and real water samples. Additionally, conventional BPA detection methods based on other composite materials have also been developed. For example, cobalt nanoparticles/nitrogen-doped carbon nanomaterials were modified on the surface of glass carbon electrodes to detect BPA. The enhancement of the device resulted in a 98% recovery rate and excellent electrocatalytic performance for BPA [56]. A self-powered molecule-imprinted photochemical sensor based on a CdSe/ZnS QDs/HOF heterostructure was used to detect BPA, and the highly sensitive and selective detection of BPA was realized [57]. A magnesium phytate-based composite modified electrode was synthesized using the hydrothermal method. Under the action of Mg^2+^, the electrode has a good response to BPA. The cost of Mg^2+^ phytate-based modified electrodes is low, and the recovery rate can reach 92.5 to 101.5% [58]. The potentiostatic deposition method was used to produce gold nanoparticles on the surface of a glass carbon electrode. These nanoparticles were then used to create a gold–ligand nanoelectrochemical sensor, which was then used to detect the presence of BPA in water. The results showed that the sensor had good anti-interference performance, but the recovery rate was only 87% [59], which is lower than the above magnesium phytate-based modified electrode.

Currently, the overall detection of BPs is relatively limited. HPLC and UPLC-MS/MS are mainly used for detection, and they can detect BP content in drinking water, human urine, biological samples, soil, leachate, and vegetable oil. Besides BPA, HPLC can be used to detect BPS, BPF, BPAF, BPAP, and BPZ. UPLC-MS/MS can also detect nearly 30 types of BPs, including those mentioned above [5,53,60]. Further, the salting-out-assisted liquid–liquid extraction HPLC-MS/MS method can detect nine types of BPs in liquid milk, namely, BPS, BPF, BPE, BPA, BPAP, BPB, BPZ, TBBPA, and TCBPA, after optimizing the traditional method. This method has a detection limit of 0.15 to 0.75 µg/kg [61].

**Table 1:** BP detection means and various detection parameters.

Detection Settings	Detectable Substances	Optimization Means	Detection Media	Advantage	Disadvantage	Recovery Rate (%)	Detection Limit	Quantification Limit	Linear Range	References
Ultra-high-performance liquid chromatography–tandem mass spectrometry	BPA, BPS, BPF, BPAF, BPAP, BPZ, etc.	-	Air, drinking water, human urine, biological samples, soil, leachate	High sensitivity, good recovery rate, and good repeatability	-	67.6~119	0.007–1.5 µg/L	0.5 µg/kg	0.1~100 µg/mL	[5,52,54,60]
High-performance liquid chromatography	BPA, BPS, BPF, BPAF, and BPAP	-	Vegetable oil, drinking water, human urine	High sensitivity and good repeatability	Difficult to achieve rapid on-site detection	90.8~103.2	0.007 mg/kg	13 µg/kg	0.003~0.7 µg/mL	[52,53,58,60]
Liquid chromatography–mass spectrometry tandem method	BPA, BPS, BPF, BPAP, etc.	Salting-out-assisted liquid–liquid extraction	Milk powder	Rapid test	-	80.8–118.1	0.15–0.75 µg/kg	0.5–2.5 µg/kg	-	[61]
Fourier change infrared spectroscopy	BPA	An amine-functionalized poly (N-vinylpyrrolidone divinylbenzene) adsorbent was prepared for the detection of BPA	Water	Low cost, good functional degree, strong selective adsorption ability, and less environmental pollution	-	99.65	0.5 mg/kg	-	0.5~3.0 µg/mL	[55]
Liquid chromatography–tandem triple quadrupole mass spectrometry	BPA	N-hexane/acetone (4:1) ultrasonic extraction three times	Dust		-	84.5~100	0.002–0.018 mg/kg	-	-	[51]
Photoelectric chemical sensor method without bias voltage	BPA	Self-powered molecularly imprinted photo/photochemical sensing based on CdSe/ZnS QD/HOF heterojunction	-	High sensitivity and high selectivity	-	-	-	-	-	[57]
Electrochemical method of carbon nanotube composites based on cobalt nanoparticles/nitrogen doping	BPA	Electrochemical method of carbon nanotube composites based on cobalt nanoparticles/nitrogen doping	-	High selectivity, good stability, and good reproducibility	-	98.4~104.6	5.0 nmol/L	0.005 µmol/L	0.01~20 (µmol/L)	[56]
Magnesium phytate-modified electrode method	BPA	Magnesium phytate-based modified electrode	-	High sensitivity, wide linear range, good repeatability, low electrode cost, and good stability	-	92.5~101.5	0.1 µmol/L	-	0.8~50 (µmol/L)	[58]
Gold nanoparticle–aptamer electrochemical sensing method	BPA	Gold nanoparticles were prepared on the surface of a glassy carbon electrode via potentiostatic deposition	Water	High sensitivity, wide linear range, good repeatability, low electrode cost, and good stability	-	87.4~110.0	10^−9^ mg/kg	10^−9^ µg/kg	10^−9^~5 × 10^−6^ µg/mL	[59]
Solid-phase extraction combined with liquid chromatography–mass spectrometry	BPA	The pH value of the sample is 2 5 mL pure methanol and 5 mL dichloromethane solution	Water		-	74.41~111.2	0.0001~0.0033 µg/L	-	0.1~100 µg/L	[62]

## 4. Potential Hazards and Risk Assessment

**Figure 4:**
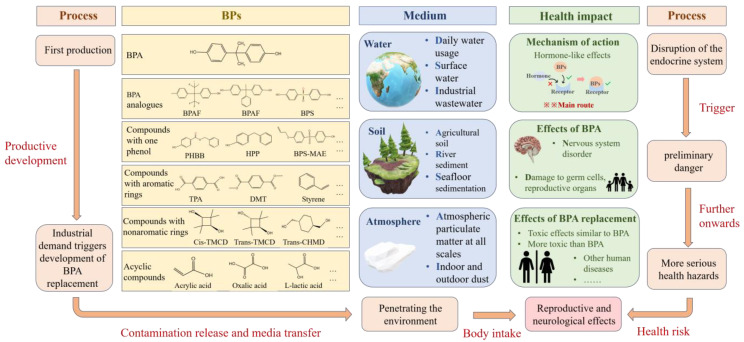
BP production, distribution, and harm to human health. The limited use of BPA has led to the emergence of BPA substitutes; BPA and its substitutes penetrate into the water, soil, and atmospheric environment; and humans are further exposed to BPs in the environment, causing adverse health effects, such as nervous, reproductive, and immune effects.

BPs can enter the human body via multiple routes, and their endocrine-disrupting effects may cause irreversible changes in both the nervous and reproductive systems (Figure 4). The hormone-like activity of BPA can disrupt the interactions between sex and thyroid hormones and their specific co-receptors, potentially resulting in neurobehavioral disorders [63]. This disrupting effect of BPA can cause neurobehavioral developmental disorders in children [64], such as attention-deficit hyperactivity disorder, internal and external behaviour problems, social and learning disorders, and ASD, as well as having an impact on the neuroimmune system of children with ASD [18,65,66]. Simultaneously, gender-specific effects of BPA on neurobehavior have been observed, with increased sensitivity in boys [4]. A study also revealed a potential causal association between BPA exposure and the risk of childhood obesity [67], with the risk of childhood obesity increasing with increasing BPA concentrations [67]. Besides direct exposure, maternal BPA exposure during pregnancy may also contribute to developmental abnormalities in offspring. Experiments have demonstrated that perinatal exposure to low doses of BPA can lead to abnormal sexual and social behaviours in offspring rats and disrupt sex-based behavioural differentiation [18]. Prenatal exposure can reduce the relative quality of thymus in male rats, and the concentration of BPA in maternal urine during pregnancy is also positively correlated with the risk of asthma in children [68]. BPA exposure can also impact the normal operation of the mammalian reproductive system. Its exposure can reduce the quality and quantity of male germ cells, indirectly leading to the abnormal development of the male reproductive system and damage to reproductive organs [31,69,70,71]. Exposure to BPA during lactation can alter the distribution of endogenous spermatocytes in the mouse testis and disrupt spermatogenic cell differentiation [72]. Moreover, the estrogenic effects of BPA may impair the quality of female oocytes, and they could be a potential factor in the development of PCOS [73]. It was found that serum BPA concentrations in infertile women were higher than those in fertile women. Therefore, high BPA concentrations inhibited the development of primordial follicles, primary follicles, and luteum. Long-term low-dose BPA exposure can cause reproductive lesions in female fallopian tubes [19,31,74]. BPA exposure can also result in abnormalities in the estrous cycle and sexual behaviour in females, lead to menstrual disorders, and increase the risk of spontaneous abortion, as well as the incidence of reproductive tract and sexual organ diseases [75]. Besides its endocrine-disrupting effects, BPA can adversely impact the reproductive system by modulating gene expression. For example, it can induce cell proliferation by affecting the cell cycle in the MCF-7 human breast cancer cell line. Moreover, exposure to low doses of BPA may precipitate the early onset of puberty in young children [63,76].

BPA alternatives have similar endocrine-disrupting effects to BPA, especially in terms of reproductive toxicity, and some can even cause stronger reproductive toxicity than BPA [10,26,77,78]. For example, BPS and BPAF are two important BPA analogues that have the potential to cause severe reproductive defects, such as germ cell apoptosis and embryo mortality. High BPS concentrations have been shown to decrease embryo viability [27,29]. The ovarian reserve may be impaired by exposure to BPS compounds, including BPA and BPS [74]. However, their mechanism of action on the toxicity of female reproduction remains unknown. The reproductive toxic effect of BPS has been preliminary investigated in several experiments, suggesting that it has the potential to cause cell cycle arrest and cell proliferation interruption in females [79]. Studies have shown that BPAF also causes reproductive toxicity in males and gonad damage in male rats, and its toxic effect may be stronger than that of BPA [33]. Therefore, future research will prioritize more in-depth and systematic toxicity studies of BPAF. Multiple BPA analogues have also been found to show cytotoxicity, genotoxicity, dioxin-like effects, and neurotoxicity [10,26,77,78]. Studies have also found a significant positive correlation between serum BPs and the risk of Sjogren’s disease [80]. Some BPs may have adverse effects on innate and adaptive immune responses. Previous studies have shown that BPA and BPAF can significantly reduce the endocytosis capacity of MDDCs. MDDCs, as the center of adaptive and innate immune response, will cause certain damage to the immune system when MDDCs is affected, and the experimental results show that the effect of BPAF is more significant [9]. The researchers have conducted further research, and the results show that when exposed to BPA and BPAF, the expression of RACK1 is significantly reduced, and the release of related cytokines is also significantly decreased [12]. RACK1 plays an important role in immune activation by regulating the balance of glucocorticoids and androgens [12]. Therefore, BPA and BPAF have significant effects on receptor binding and expression, which will lead to certain damage to the human immune system. It indicates the harm of BPs to the immune system; therefore, it is urgent to further investigate other BPs and confirm the possible harm of other BPs to the human immune system.

Although the mechanisms by which BPA affects human health are well documented, there remains a gap in research regarding the toxicity of some BPA substitutes. Considering the structural similarities between BPA and its analogues, as well as the possibility that these substitutes may pose higher toxicity, it is crucial to conduct further safety evaluations of BPA alternatives based on previous studies on BPA [81].

There is a mature health assessment for BPA that can be carried out using a risk assessment model and exposure level. Currently, daily intake (EDI) can be used to evaluate the levels of BPA exposure in different environmental media [47,82]. Simultaneously, the hazard quotient (HI) is used to assess the health risk of BPA, with HI > 1 indicating a carcinogenic risk [82]. The health risk assessment methods of BPA are not special or unique; however, due to the complexity of BPA, the health risks of BPA and its metabolites in vivo are a major concern. After BPA, BPA alternatives will become a new focus due to the continuous expansion of BP substances. However, the majority of BPA alternatives lack certain computational parameters, making it difficult to calculate their health risks. Recently, BPA has been found to be the main chemical substance in BPs, and the proportion of BPA in BPs is higher than that of other BPA alternatives. Therefore, assessing the health risks of BPA has become a priority. In this study, the health risk was evaluated based on BPA data obtained from the literature. The parameters used are shown in Table 2, and the evaluation was carried out using Formulas (1) and (2) [43]:

(1) $$EDI=\frac{\left(C\times IR\right)}{BW}$$

(2) $$HI=\sum \frac{EDI}{RfD}$$

**Figure 5:**
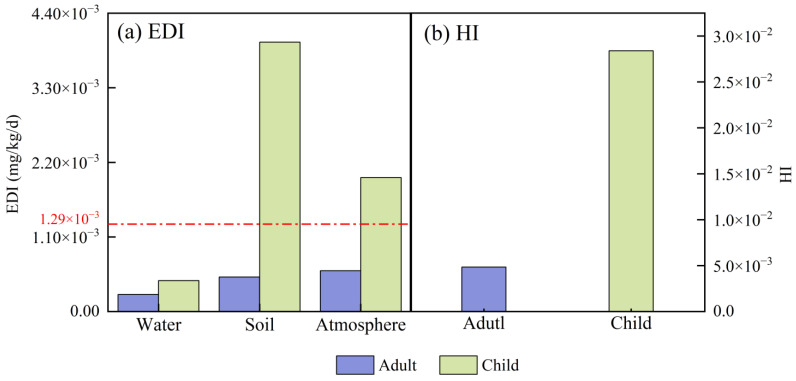
EDI level and HI assessment analyses of BPs in adults and children (notes: EDI_water_ × 10^8^) [15,22,39,41,42,44,46,47,48,49]. Using CNKI as the search database and BPs, water, soil, and atmosphere as the search keywords, the concentration data of BPs in the three media were retrieved, and the historical concentration data were used to calculate the EDI and HI values according to the empirical formula given by EPA, so as to evaluate the health risks in each environment. The red dotted line is the average exposure level for the whole environment.

As shown in Figure 5, the EDI exposure level shows the phenomenon of water < atmosphere < soil. BPs are not easily soluble in water, and their exposure level in water is relatively low. The EDI value of soil is higher than the average exposure level of the whole environment (1.29 × 10^−3^), followed by the atmosphere, suggesting that more attention needs to be paid to BPA pollution in soil and the atmosphere. The average value of the overall environment needs to be lower than the present calculated average, as the EDI value in the water environment is overestimated in the calculation (1.29 × 10^−3^). The EDI is consistently higher in children than in adults, which may be attributed to the comparatively low body weight of children. Further, HI < 1, suggesting that the current BPA level poses no carcinogenic risk to children or adults and can be disregarded. Children are at a higher health risk than adults, as evidenced by their health risk being six times greater than that of adults (HI children (0.03) > HI adults (0.005)). This may be attributed to the immaturity of children’s bodies and their compromised metabolisms, which can increase their susceptibility to pollutants [83,84]. After analyzing the data, it is evident that the current levels of environmental exposure and associated health risks are not alarmingly high; however, it is important to understand that children are still susceptible to these health risks.

Most BPA alternatives have not undergone comprehensive safety assessments. Current testing indicates that the environmental concentrations of other BPs are much lower than those of BPA. Thus, the current exposure levels and associated health risks of BPA alternatives will likely be considerably lower than those recently estimated for BPA. However, considering the continuous expansion of BP applications and alternatives with a higher toxicity than BPA, the exposure levels and health risks of BPA alternatives should be continuously considered. Potential risk prediction models have been developed for several chemicals to predict the potential risk of BPs without experimental data [85,86]. For example, Hong et al. [86] predicted the activity of 29 types of bio-based platform chemicals in estrogen receptors using computer models, and they found that approximately 15 related species were expected to be estrogen conjugations. Karrer et al. [85] used a model to estimate human BP exposure levels, and they found that diet and tea polyphenols may be the most important sources of BP exposure. However, there is still a considerable lack of experimental data to substantiate the potential health risk assessments for BPA alternatives. Thus, it remains crucial to develop effective health risk assessments for these BPA alternatives, relying on future field observation data.

## 5. Suggestions for Future Prevention and Control Countermeasures

In many countries, the prevention and control of the impact of BPs on human health have become issues that require immediate solutions, especially for BPA. Table 3 summarizes the restrictions placed on BPA in several countries and regions; these restrictions focus on limiting the use of BPA in infant products, which is consistent with the above conclusions regarding children’s susceptibility to BPs. The Toxin-Free Keiki measure, recently announced by the U.S. state of Hawaii, prevents the use of BPA in reusable food or beverage containers for children as young as three years old. Further, Illinois prohibits the use of BPA in commercial or bank record papers [87]. In 2022, China listed BPA as a pollutant with high pollution and environmental risks [88]. California also contemplated the inclusion of BPA on the list of carcinogens [89].

Despite the progress in current BP research, problems remain in terms of pollution prevention efforts. First, a major issue is pollution emissions. Due to the physical and chemical properties of BPA, its main source is solid waste combustion [25]. Therefore, it is necessary to limit the emissions of major emission points, control the pollution emissions of BPA from the source, avoid the direct incineration of plastic waste, and properly handle the recycling and disposal of plastic products. Considering the structural similarities between BPA and its substitutes [29], they may share common emission sources. Therefore, it is crucial to monitor the emissions of other BPs during the combustion of solid waste and to develop comprehensive testing protocols. This approach will enhance the detection of BPA and its replacements in various environments, particularly emphasizing atmospheric and soil conditions. BPs in the atmosphere are mainly affected by direct emissions. Simultaneously, BP pollution in soil is related to agricultural activities, which differ according to the farming method and region, resulting in great differences in soil pollution [46,47]. It is necessary to formulate management measures according to local conditions in order to meet agricultural needs and reduce the pollution of soil by BPs. First, it is crucial to establish more efficient and stable BPA substitutes, enhance the efficacy of BP applications, and reduce their entry into the environment to protect production and life. Second, focus should be placed on advancing detection technologies for BPA and its alternatives and assessing the safety of these compounds. Currently, the primary methods for detecting BPA alternatives include HPLC, LC-MS/MS, and UPLC-MS/MS [5,52,53,54,60,62]. Recently, new detection methods for BPA have been developed, in addition to traditional detection methods [56,57,58,59]. To enhance the efficiency of detecting BPA substitutes, efforts should focus on expanding the detection capabilities of emerging technologies, building on the existing methods for BPA analysis. This approach aims to acquire more comprehensive and accurate data on BP compounds. Moreover, safety assessments of BPs need to be conducted. Currently, safety assessments of BPs are carried out using computer simulations [85,86], but there is still a lack of field observational and experimental data for comparative verification. Therefore, it is necessary to strengthen the combination of theoretical simulation and field observation results in the future. To ensure efficient, low-toxicity, safe, and stable production and to protect life, it is crucial to verify and fully investigate the safety evaluations of BPs, focusing on examining BPA alternatives. Third, it is necessary to formulate relevant policies and regulations. In many regions, regulations for the use of BPA have been established, resulting in the restriction of its use in specific products [87,88,89,90,91]. However, due to the expansion of industrial demand and the continuous development of BPA alternatives, new BPs are emerging in an endless stream [10,26,77,78]. Therefore, along with the safety assessments of BPA, it is essential to broaden restrictions on other toxic BP compounds. Simultaneously, industry-specific standards for BP limits must be established, particularly for sectors such as plastics, including products such as baby pacifiers and paper supplies. This requires increasing regulatory oversight, developing corresponding policies, controlling industrial pollution emissions, and ensuring the proper recycling and disposal of solid waste.

## 6. Conclusions

BPs have attracted attention due to their potential health impacts. However, despite recent progress in environmental and biological aspects, challenges remain, including rapid on-site detection methods, more refined detection techniques, and comprehensive safety assessments of BPA alternatives. These gaps highlight the necessity for further research and new developments. The second issue is the absence of safety assessments of BPA alternatives. This necessitates in-depth studies of BPs’ mechanisms to better identify their health risks and develop appropriate preventative and control approaches. This study offers the following directions for future research. First, BP detection range should be expanded upon, focus should be given to soil and atmosphere pollution, a complete BP detection list should be developed, toxic BPA alternatives should be prevented from entering the environment and biological systems, and an understanding of the pollution and distribution characteristics of related contaminants should be facilitated. Second, future research should prioritize the development of more advanced and efficient detection technologies for BPs to facilitate rapid and accurate assessments. Third, it is crucial to explore the mechanisms underlying BPs’ effects and conduct comprehensive safety evaluations to build a rigorous health assessment system. This will ensure that BPs meet industrial requirements without causing detrimental biological health effects. Addressing these research areas is crucial for managing the challenges caused by BP pollution and protecting environmental and human health.

## Figures and Tables

**Table 2:** Relevant parameters.

Parameter	Age	Reference Data
Rfd (µg/g/d)	-	0.05
IR_Water_ (L/d)	Adult	1.85
	Child	0.86
IR_Soil_ (mg/d)	Adult	100
	Child	200
IR_Atmosphere_ (m^3^/d)	Adult	12.8
	Child	7.8
BW (kg)	Adult	58.6
	Child	15

**Table 3:** The relevant provisions of international organizations on PAEs.

Region/Organization	Bill	Stipulation	Reference
US. Hawaii	HB139(HD1) Toxin-Free Keiki Bill	BPA is banned from reusable food or drink containers for children as young as three years old	[87]
US. Illinois	HB2076 Bill	BPA is prohibited from being used in commercial or bank record paper	[87]
US. New York	S1076 Bill	No BPA in toys or in cans and other containers containing liquids or beverages intended for children aged three years or younger	[87]
US. New York	S3056 Bill	BPA is prohibited in childcare products for children aged three years or younger	[87]
EU	2011/8/EU	The chemical BPA is prohibited from being used in the production of baby bottles, requiring that all plastic materials that come into contact with food have no more than 0.6 mg/kg of BPA allowed to migrate	[90]
Food and Drug Administration (FDA)	2022-14682	The authorized use of BPA as a food additive is revoked and limited to establish a maximum limit of 0.5 ng/kg in food	[91]
CN, Ministry of Ecology and Environment of the People’s Republic of China	Comprehensive List of Environmental Protection (2021 version)	BPA is listed as a “high-pollution, high-environmental-risk” product	[88]

## Data Availability

The original contributions presented in this study are included in the article/Supplementary Material. Further inquiries can be directed to the corresponding author(s).

## Supplementary Materials

The following supporting information can be downloaded at the following: https://www.mdpi.com/article/10.3390/toxics13020109/s1. Table S1: The molecular formulae of each BP and their associated physical parameters; Table S2: Distribution coefficients for each of the BPs, simulated using EPI; Table S3: Concentration levels of common BPs in water, soil, and atmospheric media worldwide.

## References

[1] Yu Y., Wang S., Yu P., Wang D., Hu B., Zheng P., Zhang M. (2024). A Bibliometric Analysis of Emerging Contaminants (ECs) (2001−2021): Evolution of Hotspots and Research Trends. Sci. Total Environ..

[2] Darbre P. (2018). Overview of Air Pollution and Endocrine Disorders. Int. J. Gen. Med..

[3] Buoso E., Masi M., Racchi M., Corsini E. (2020). Endocrine-Disrupting Chemicals’ (EDCs) Effects on Tumour Microenvironment and Cancer Progression: Emerging Contribution of RACK1. Int. J. Mol. Sci..

[4] Tian Y., Zhou X., Miao M., Li D., Wang Z., Li R., Liang H., Yuan W. (2018). Association of Bisphenol A Exposure with LINE-1 Hydroxymethylation in Human Semen. Int. J. Environ. Res. Public Health.

[5] Wang L. (2022). The Pollution Characteristics and Bioaccumulation of BPs in Multiple Media at Longyearbyen, Arctic. Master’s Thesis.

[6] Qiao Y., Yan Z., Feng C., Wang J., Bai Y., Wu F. (2022). Research Focus Analysis of Endocrine Disrupting Chemicals (EDCs) Based on Bibliometrics. Res. Environ. Sci..

[7] Schneider M., Pons J.-L., Labesse G., Bourguet W. (2019). In Silico Predictions of Endocrine Disruptors Properties. Endocrinology.

[8] Yang J. (2024). Endocrine Disrupting Effects of Typical Bisphenols and their Ecological Risks in the Sediments of Taihu Laike. Master’s Thesis.

[9] Švajger U., Dolenc M.S., Jeras M. (2016). In Vitro Impact of Bisphenols BPA, BPF, BPAF and 17β-Estradiol (E2) on Human Monocyte-Derived Dendritic Cell Generation, Maturation and Function. Int. Immunopharmacol..

[10] Qiu W., Liu S., Chen H., Luo S., Xiong Y., Wang X., Xu B., Zheng C., Wang K.-J. (2021). The Comparative Toxicities of BPA, BPB, BPS, BPF, and BPAF on the Reproductive Neuroendocrine System of Zebrafish Embryos and Its Mechanisms. J. Hazard. Mater..

[11] Lambré C., Barat Baviera J.M., Bolognesi C., Chesson A., Cocconcelli P.S., Crebelli R., Gott D.M., Grob K., Lampi E., EFSA Panel on Food Contact Materials, Enzymes and Processing Aids (CEP) (2023). Re-evaluation of the Risks to Public Health Related to the Presence of Bisphenol A (BPA) in Foodstuffs. EFSA J..

[12] Buoso E., Kenda M., Masi M., Linciano P., Galbiati V., Racchi M., Dolenc M.S., Corsini E. (2021). Effects of Bisphenols on RACK1 Expression and Their Immunological Implications in THP-1 Cells. Front. Pharmacol..

[13] Masi M., Racchi M., Travelli C., Corsini E., Buoso E. (2021). Molecular Characterization of Membrane Steroid Receptors in Hormone-Sensitive Cancers. Cells.

[14] Liu Z., Zhao X., Zhang Y., Li Y., Qiu J., Mu X., Jiang J., Qian Y. (2023). Advances in potential impacts of bisphenol A and its alternatives on gut-brain regulation and involved mechanisms. Asian J. Ecotoxicol..

[15] Xiong S., Wang X., Luo W., Ma Y., Lin Y., Wang M., Zheng J. (2021). Spatia Distribution, Ecological Risk and Industry-Dependence of Endocrine Disrupting Chemicals in the Beijiang River, South China. Environ. Chem..

[16] Wang Q., Feng Q., Zhu X. (2023). Determination of bisphenols in sediment by accelerated solvent extraction and solid-phase extraction purification coupled with ultra performance liquid chromatography-tandem mass spectrometry. Chin. J. Chromatogr..

[17] Li C., Li M., Zhu A., Liu G., Liu P. (2025). Distribution and ecological risk assessment of bisphenols in Fuhe River and Baiyang Lake. J. Environ. Health.

[18] Qin Y., Jian B. (2016). Impact of Bisphenol A Pollution on Nervous Immune System of Children with Autism Spectrum Disease. Environ. Sci. Manag..

[19] Chen W., Lau S.-W., Fan Y., Wu R.S.S., Ge W. (2017). Juvenile Exposure to Bisphenol A Promotes Ovarian Differentiation but Suppresses Its Growth—Potential Involvement of Pituitary Follicle-Stimulating Hormone. Aquat. Toxicol..

[20] Usman A., Ahmad M. (2016). From BPA to Its Analogues: Is It a Safe Journey?. Chemosphere.

[21] Zhang T., Xue J., Gao C., Qiu R., Li Y., Li X., Huang M., Kannan K. (2016). Urinary Concentrations of Bisphenols and Their Association with Biomarkers of Oxidative Stress in People Living Near E-Waste Recycling Facilities in China. Environ. Sci. Technol..

[22] Gibson R., Durán-Álvarez J.C., Estrada K.L., Chávez A., Jiménez Cisneros B. (2010). Accumulation and Leaching Potential of Some Pharmaceuticals and Potential Endocrine Disruptors in Soils Irrigated with Wastewater in the Tula Valley, Mexico. Chemosphere.

[23] Ji Z., Liu J., Sakkiah S., Guo W., Hong H. (2021). BPA Replacement Compounds: Current Status and Perspectives. ACS Sustain. Chem. Eng..

[24] US Environmental Protection Agency (EPA) (2014). Bisphenol A Alternatives in Thermal Paper. https://www.epa.gov/sites/default/files/2015-08/documents/bpa_final.pdf.

[25] Li J., Wang G. (2015). Airborne Particulate Endocrine Disrupting Compounds in China: Compositions, Size Distributions and Seasonal Variations of Phthalate Esters and Bisphenol A. Atmos. Res..

[26] Chen D., Kannan K., Tan H., Zheng Z., Feng Y.-L., Wu Y., Widelka M. (2016). Bisphenol Analogues Other Than BPA: Environmental Occurrence, Human Exposure, and Toxicity—A Review. Environ. Sci. Technol..

[27] Shi Z., Qin H., Gao J., Qu G., Jiang G. (2024). The effect of bisphenols on the secretion of exosomes. Environ. Chem..

[28] Pastor-Belda M., Bastida D., Campillo N., Pérez-Cárceles M.D., Motas M., Viñas P. (2016). A Study of the Influence on Diabetes of Free and Conjugated Bisphenol A Concentrations in Urine: Development of a Simple Microextraction Procedure Using Gas Chromatography–Mass Spectrometry. J. Pharm. Biomed. Anal..

[29] Chen Y., Shu L., Qiu Z., Lee D.Y., Settle S.J., Que Hee S., Telesca D., Yang X., Allard P. (2016). Exposure to the BPA-Substitute Bisphenol S Causes Unique Alterations of Germline Function. PLoS Genet.

[30] US Environmental Protection Agency (EPA) EPI SuiteTM-Estimation Program Interface. https://www.epa.gov/tsca-screening-tools/epi-suitetm-estimation-program-interface.

[31] Liu J., Jin Y., Wei Q., Jin Z., Wei D., Jin Y. (2022). Research progress on reproductive toxicity and reproductive system tumors induced by environmental endocrine disrupting chemicals. J. Environ. Occup. Med..

[32] Devi T., Saleh N.M., Kamarudin N.H.N., Roslan N.J., Jalil R., Hamid H.A. (2023). Efficient Adsorption of Organic Pollutants Phthalates and Bisphenol A (BPA) Utilizing Magnetite Functionalized Covalent Organic Frameworks (MCOFs): A Promising Future Material for Industrial Applications. Ecotoxicol. Environ. Saf..

[33] Zhu X., Cao L., Liu Y., Tang X., Miao Y., Zhang J., Chen J. (2024). Genotoxicity of Bisphenol AF in Rats: Detrimental to Male Reproductive System and Probable Stronger Micronucleus Induction Potency than BPA. J. Appl. Toxicol..

[34] Fu P., Kawamura K. (2010). Ubiquity of Bisphenol A in the Atmosphere. Environ. Pollut..

[35] Jiang X., Rotily L., Villermaux E., Wang X. (2022). Submicron Drops from Flapping Bursting Bubbles. Proc. Natl. Acad. Sci. USA.

[36] Wang S., Rao Z., Guo F., Liu C., Zhan N., Wang Y., Peng J., Yang H. (2021). Occurrence Characteristics and Health Risk Assessment of Endocrine Disrupting Chemicals in Groundwater in Wuxi-Changzhou. Environ. Sci..

[37] Wang P. (2020). Study on Migration and Transformation of Representative Endocrine Disrupt Chemicals during Reclaimed Water Infltrating into Groundwater. Ph.D. Thesis.

[38] Ju X., Gao Z., Zheng W., Zhang Q. (2024). Identification and Derivation of Emerging Contaminants in the Roof Rainwater Confluence. Environ. Sci..

[39] Zhao B., Tan X., Xue M., Lu J., Xu D., Yang R., Zhang L., Gou W. (2023). Pollution Status and Distribution Characteristics of Bisphenols in Rivers of Guangyuan City. Environ. Monit. Forewarning.

[40] Dueñas-Moreno J., Mora A., Cervantes-Avilés P., Mahlknecht J. (2022). Groundwater Contamination Pathways of Phthalates and Bisphenol A: Origin, Characteristics, Transport, and Fate – A Review. Environ. Int..

[41] Kumawat M., Sharma P., Pal N., James M.M., Verma V., Tiwari R.R., Shubham S., Sarma D.K., Kumar M. (2022). Occurrence and Seasonal Disparity of Emerging Endocrine Disrupting Chemicals in a Drinking Water Supply System and Associated Health Risk. Sci Rep.

[42] Chen M., Guo M., Liu D., Li J., Zhang S., Shi L. (2017). Occurrence and distribution of typical endocrine disruptors in surface water and sediments from Taihu Lake and its tributaries. Chin. Environ. Sci..

[43] Ma B., Wang L., Tao W., Liu M., Zhang P., Zhang S., Li X., Lu X. (2020). Phthalate Esters in Atmospheric PM2.5 and PM10 in the Semi-Arid City of Xi’an, Northwest China: Pollution Characteristics, Sources, Health Risks, and Relationships with Meteorological Factors. Chemosphere.

[44] Yan Y., Huang Q., Mou J., Luo W. (2017). Bisphenol A secondary pollution in barreled drinking water and its exposure assessment. Pract. Prev. Med..

[45] Xu Y., Hu A., Li Y., He Y., Xu J., Lu Z. (2021). Determination and Occurrence of Bisphenol A and Thirteen Structural Analogs in Soil. Chemosphere.

[46] Ye Z. (2021). Studies on the Pollution Distribution of Typical Bisphenol Compounds in Soil and Earthworms and Toxicity Effects. Master’s Thesis.

[47] Zhang Y. (2022). Research on Pollution Characteristics and Health Risks of Bisphenols in Soil at National Scale. Master’s Thesis.

[48] Teil M.-J., Moreau-Guigon E., Blanchard M., Alliot F., Gasperi J., Cladière M., Mandin C., Moukhtar S., Chevreuil M. (2016). Endocrine Disrupting Compounds in Gaseous and Particulate Outdoor Air Phases According to Environmental Factors. Chemosphere.

[49] Liu C., Yang Y., Yu J., Xu J. (2019). Investigation and Environmental Risk Assessment of Octylphenol, Nonylphenol and Bisphenol A in the Sediment of Xiangjiang River in Zunyi City.

[50] Loganathan S.N., Kannan K. (2011). Occurrence of Bisphenol A in Indoor Dust from Two Locations in the Eastern United States and Implications for Human Exposures. Arch. Env. Contam. Toxicol..

[51] Liu W., Wang Y., Liu Y., Sun Y., Wu M., Ma J. (2019). Comparative assessment of human exposure to phthalate esters and bisphenol A from different indoor dust. J. Shanghai Univ. Nat. Sci..

[52] Wu W., Guan C., Wang G., Wang Z., Chen Z., Li L., Feng D. (2022). A Study on Determination of 8 Kinds of Bisphenols in Packaged Drinking Water by High Per-Formance Liguid Chromatography-Tandem Mass Spectrometry. China Meas. Test.

[53] Liu Y., Jiang W., Hu R., Wu X. (2022). Study of the Method for the Determination of Bisphenol a in Vegetable Oil by High Performance Liquid Chromatography. Sci. Technol. Cereals Oils Foods.

[54] Ling Y., Liu Z. (2022). Determination of 14 Environmental Hormones in Workplace Air by Ultra-High Performance Liquid Chromatography-Tandem Mass Spectrometry. PTCA Part B Chem. Anal..

[55] Zhang J., Cui Y., He L., Zhang Y., Meng X., Zhang S. (2023). Modification of P(NVP-DVB) and its application in the detection of BPA in aqueous solution. Fine Chem..

[56] Tang J., Cheng X., Cui X., Zhu Y., Fan J., Liu T., Zheng S. (2023). Electrochemical Detection of Bisphenol A Based on Co Nanoparticles/N-Doped Carbon Nano-Tubes Composites. Chin. J. Anal. Lab..

[57] Zhang X., Yu L., Bai B., Guo C., Zhang J., Yang Y. (2022). A self- powered molecular imprinting photoelectrochemical sensor based on CdSe ZnS QDs/HOFs heterojunction for the detection of bisphenol A. Proceedings of the Chinese Society of Food Science and Technology 19th Annual Meeting Paper Abstracts Col-Lection.

[58] Zhang Y., Li Y., Yu H., Wang H. (2023). Preparation of magnesium phytate-based modified electrode and its detection for bisphenol A. Chin. J. Anal. Lab..

[59] Meng X., Zhang Y., Liu J., Tan F. (2023). Gold nanoparticles-aptamer electrochemical sensor for detection of bisphenol A in environmental waters. Environ. Chem..

[60] Wu W., Guan C., Wang G., Wang Z., Chen Z., Li L., Chen Q., Cao J., Feng D. (2023). Determination of 8 Kinds of Bisphenols in Urineby Solid Phase Extraction Combined with High Performance Liquid Chromatography-Tandem Mass Spectrometry. China Meas. Test.

[61] Jiang K., Zhang H., Cao H., Wang J., Zhou X., Li X. (2022). Salting out Assisted Liquid Liquid Extraction Coupled with HPLC-MS/MS for Determination of 13 Kinds of Bisphenols and Alkyl Phenol Compounds in Liquid Dairy Products. Food Mach..

[62] Wang T., Xu Q., Li J., Li J., Tong W. (2023). Simultaneous determination of 9 alkyl phenols and bisphenol A in water by SPE combined with LC-MS/MS. Water Wastewater Eng..

[63] Huang Y., Zhang W., Wang R., Su X. (2022). Advances on pollution status and endocrine disrupting effects of bisphenols. Asian J. Ecotoxicol..

[64] Li Y., Jiao Z., Zhang X., Fu W., Wang L., Gong H., Jiang G. (2023). Progress in the treatment technologies toward endocrine disrupter bisphenol A. Environ. Chem..

[65] Hong S., Hong Y., Kim J., Park E., Shin M., Kim B., Yoo H., Cho I., Bhang S., Cho S. (2013). Bisphenol A in Relation to Behavior and Learning of School-age Children. Child Psychol. Psychiatry.

[66] Xie N., Wang H., Wang S. (2022). Research progress on the effects of bisphenol A exposure on children’s health. Matern. Child Health Care China.

[67] Kim K.Y., Lee E., Kim Y. (2019). The Association between Bisphenol A Exposure and Obesity in Children—A Systematic Review with Meta-Analysis. Int. J. Environ. Res. Public Health.

[68] Tian H., Shan L., Cui K., Ru S. (2022). Pollution status and adverse effects of bisphenols on early life. Asian J. Ecotoxicol..

[69] Wu Y., Lu X., Ren Y., Sun S. (2022). The effect of environmental endocrine disruptoe bisphenol A on female reproductive system. J. Zunyi Med. Univ..

[70] Xie Y., Zhu Y., Tao F. (2019). Childhood and adolescent obesity and maternal phthalate exposure during pregnancy. Chin. J. Sch. Health.

[71] Tian J., Ding Y., She R., Ma L., Du F., Xia K., Chen L. (2017). Histologic Study of Testis Injury after Bisphenol A Exposure in Mice: Direct Evidence for Impairment of the Genital System by Endocrine Disruptors. Toxicol. Ind. Health.

[72] Huang X., Ling Y., Zhao C., Chen J., Zhang J., Liu Y., Xie M. (2021). Mechanism of meiotic arrest of spermatogenic cells in testis of mice exposed to bisphenol A during lactation. J. Environ. Occup. Med..

[73] Wang Y., Zhu Q., Dang X., He Y., Li X., Sun Y. (2017). Local Effect of Bisphenol A on the Estradiol Synthesis of Ovarian Granulosa Cells from PCOS. Gynecol. Endocrinol..

[74] Zhang N., Zhao Y., Zhai L., Bai Y., Wei W., Sun Q., Jia L. (2024). Urinary Concentrations of Bisphenol A and Its Alternatives: Potential Predictors of and Associations with Antral Follicle Count among Women from an Infertility Clinic in Northern China. Environ. Res..

[75] Zhou Y., Lou R. (2019). The effect of bisphenol A on the reproductive system and its mechanism. Matern. Child Health Care China.

[76] Li Y., Zhang H., Kuang H., Fan R., Cha C., Li G., Luo Z., Pang Q. (2018). Relationship between Bisphenol A Exposure and Attention-Deficit/ Hyperactivity Disorder: A Case-Control Study for Primary School Children in Guangzhou, China. Environ. Pollut..

[77] Wei J., He Z., Wang C., Hu Y., Wang C., Cao H., Cao M., Liang Y. (2022). Progress in Reproductive Toxicity and Human Reproductive Health Risk of Bisphenol A Analogues. Asian J. Ecotoxicol..

[78] Pelch K., Wignall J.A., Goldstone A.E., Ross P.K., Blain R.B., Shapiro A.J., Holmgren S.D., Hsieh J.-H., Svoboda D., Auerbach S.S. (2019). A Scoping Review of the Health and Toxicological Activity of Bisphenol A (BPA) Structural Analogues and Functional Alternatives. Toxicology.

[79] Yu M., Yang Z., Zhou Y., Guo W., Tian L., Zhang L., Li X., Chen J. (2024). Mode of Action Exploration of Reproductive Toxicity Induced by Bisphenol S Using Human Normal Ovarian Epithelial Cells through ERβ-MAPK Signaling Pathway. Ecotoxicol. Environ. Saf..

[80] Liao K., Zhao Y., Qu J., Yu W., Hu S., Fang S., Zhao M., Jin H. (2024). Association of Serum Bisphenols, Parabens, and Triclosan Concentrations with Sjögren Syndrome in the Hangzhou, China Population. Sci. Total Environ..

[81] Gély C.A., Lacroix M.Z., Morin M., Vayssière C., Gayrard V., Picard-Hagen N. (2021). Comparison of the Materno-Fetal Transfer of Fifteen Structurally Related Bisphenol Analogues Using an Ex Vivo Human Placental Perfusion Model. Chemosphere.

[82] Dehdashti B., Nikaeen M., Amin M.M., Mohammadi F. (2023). Health Risk Assessment of Exposure to Bisphenol A in Polymeric Baby Bottles. Env. Health Insights.

[83] Li H., Yang Z., Xu X., Liu Z., Xian J., Yang S., Zhang C. (2022). Pollution Characteristics and Health Risk Assessment of Phthalate Esters in Household Dust in Chengdu, China. Hum. Ecol. Risk Assess. Int. J..

[84] Zhang K., Feng L., Zhang P., Zhang D., Li X. (2024). Pollution Characteristics and Health Risk Assessment of Phthalate Esters (PAEs) in Soils of Typical Agricultural Areas in Qingdao. Environ. Chem..

[85] Karrer C., Andreassen M., Von Goetz N., Sonnet F., Sakhi A.K., Hungerbühler K., Dirven H., Husøy T. (2020). The EuroMix Human Biomonitoring Study: Source-to-Dose Modeling of Cumulative and Aggregate Exposure for the Bisphenols BPA, BPS, and BPF and Comparison with Measured Urinary Levels. Environ. Int..

[86] Hong H., Harvey B., Palmese G., Stanzione J., Ng H., Sakkiah S., Tong W., Sadler J. (2016). Experimental Data Extraction and in Silico Prediction of the Estrogenic Activity of Renewable Replacements for Bisphenol A. Int. J. Environ. Res. Public Health.

[87] Several States in the United States Have Issued New Regulations to Control Bisphenol A. https://bz.hgcm.cn/zggmsb/2019-03-25/details.html?edition=2&details=12.

[88] Ministry of Ecology and Environment of the People’s Republic of China Comprehensive List of Environmental Protection (2021 Edition). https://www.mee.gov.cn/ywdt/hjywnews/202111/t20211103_959023.shtml.

[89] Chau K. Request for Relevant Information on the Carcinogenicity of Bisphenol A (BPA). https://oehha.ca.gov/proposition-65/crnr/request-relevant-information-carcinogenicity-bisphenol-bpa.

[90] Program H.F. Bisphenol A (BPA). Food and Drug Administration (FDA) 2024. https://www.fda.gov/food/food-packaging-other-substances-come-contact-food-information-consumers/bisphenol-bpa.

[91] Food and Drug Administration, US Department of Health and Human Services
Food and Drug Administration. https://www.federalregister.gov/documents/2022/07/11/2022-14682/environmental-defense-fund-maricel-maffini-breast-cancer-prevention-partners-clean-water-actionclean.

