# Supplementary material for: Contamination Characterization, Toxicological Properties, and Health Risk Assessment of Bisphenols in Multiple Media: Current Research Status and Future Perspectives
Source: Toxics. 2025 Jan 29;13(2):109. doi: 10.3390/toxics13020109 (PMC11860711; doi:10.3390/toxics13020109)
Supplement: Supplementary file 1 [file toxics-13-00109-s001.zip › toxics-3404053-supplementary.pdf]

Contamination Characterization, Toxicological Properties, and Health Risk Assessment of Bisphenols in Multiple Media: Current Research Status and Future Perspectives

Fangyun Long [1], Yanqin Ren [1,*], Fang Bi [1,*], Zhenhai Wu [1], Haijie Zhang [1], Junling Li [1], Rui Gao [1], Zhengyang Liu [1], Hong Li [1]

[1] State Key Laboratory of Environmental Criteria and Risk Assessment, Chinese Research Academy of Environmental Sciences, Beijing 10012, China

Corresponding author E-mail: renyq@craes.org.cn (Yanqin Ren); bifang@craes.org.cn (Fang Bi)

Table S1 The molecular formulae of each BP and their associated physical parameters are presented herewith.

Type	Chemical compound	CAS	Molecular formula	Molecular mass	Densities (g/cm^3)	Boiling (℃)	Melting point (℃)	Water solubility (mg/L)
Bisphenol A	BPA	80-05-7	$C_{15}H_{16}O_2$	228.29	1.1	363.54	131.76	172.7
BPA analogues	Bisphenol F (BPF)	620-92-8	$C_{13}H_{12}O_2$	200.24	1.2	351.92	128.72	542.8
	Bisphenol E (BPE)	2081-08-5	$C_{14}H_{14}O_2$	214.26	1.2	356.53	129.8	265
	Bisphenol B (BPB)	77-40-7	$C_{16}H_{18}O_2$	242.32	1.1	375.14	139.43	29.23
	Bisphenol C (BPC)	79-97-0	$C_{17}H_{20}O_2$	256.35	1.1	386.74	152.11	7.459
	Bisphenol AP (BPAP)	1571-75-1	$C_{20}H_{18}O_2$	290.36	1.2	436.09	182.2	3.758
	Bisphenol Z (BPZ)	843-55-0	$C_{18}H_{20}O_2$	268.36	1.2	411.74	161.18	1.472
	Bisphenol AF (BPAF)	1478-61-1	$C_{15}H_{10}F_6O_2$	336.24	1.4	347.16	125.78	4.302
	Bisphenol S (BPS)	80-09-1	$C_{12}H_{10}O_4S$	250.27	1.4	422.52	176.41	3518
Compounds with one phenol	3,5-Dihydroxy-4-(butylbenzyl)benzoic acid (PHBB)	94-18-8	$C_{14}H_{12}O_3$	228.24	1.1799	170	109	92
	Bisphenol P	101-53-1	$C_{13}H_{12}O$	184.23	1.012	198	79	99.99
	p-(tert-butyl)-pheno	98-54-4	$C_{10}H_{14}O$	150.22	0.908	236	96	8700
	Gallic acid	149-91-7	$C_7H_6O_5$	170.12	1.694	259.73	251	12000
	BPS-MAE	97042-18-7	$C_{15}H_{14}O_4S$	290.33	1.271	495.8	168	5.95
Compounds with aromatic rings	terephthalic acid (TPA)	100-21-0	$C_8H_6O_4$	166.13	1.58	214.32	>300	17
	dimethyl terephthalate (DMT)	120-61-6	$C_{10}H_{10}O_4$	194.18	1.29	288	140	-
	Styrene	100-42-5	C_8H_8	104.15	-	145	-31	300
Compounds with nonaromatic	Limonene	138-86-3	$C_{10}H_{16}$	136.23	-	170	-84	10

rings								
	Trans-CHDM	3236-48-4	$C_8H_{16}O_2$	144.21	1.02	283	61	-
	Cis-TMCD	3039-96-1	$C_8H_{16}O_2$	144.21	-	-	-	-
Acyclic compounds	Acrylic acid	79-10-7	$C_3H_4O_2$	72.06	1.051	139	13	-
	L-lactic acid	50-21-5	$C_3H_6O_3$	90.08	1.209	122	18	-
	Methacrylic acid	79-41-4	$C_4H_6O_2$	86.09	1.015	163	12	-

Table S2 The distribution coefficients for each of the BPs were simulated using the EPI.

Compound	$LogK_{ow}$	$LogK_{oc}$	$LogK_{oa}$
BPA	3.32	4.876	12.747
BPF	2.91	4.472	12.582
BPE	3.19	4.699	12.74
BPB	4.13	5.174	13.432
BPC	4.74	5.313	14.079
BPAP	4.86	6.261	15.503
BPZ	5.00	5.784	14.413
BPAF	4.47	6.205	12.104
BPS	1.65	3.882	14.607
PHBB	3.7	3.509	11.483
HPP	3.54	4.071	9.160
p-(tert-butyl)-pheno	3.42	3.109	7.623
Gallic acid	0.86	1.563	18.002
BPS-MAE	3.05	3.482	13.133
TPA	1.76	1.899	12.050
DMT	1.66	1.491	4.511
Trans-CHDM	3.33	1.219	9.567
Cis-TMCD	1.27	0.805	6.159
Acrylic acid	0.44	0.349	5.170
L-lactic acid	-0.65	-0.654	4.758
Methacrylic acid	0.99	0.670	5.730

The relevant parameters are simulated by EPI software

Table S3 Concentration levels of common BPs in water, soil, and atmospheric media worldwide

medium	Time	Point	Detectable level								Data sources
			BPA	BPS	BPF	BPZ	BPAP	BPAF	BPP	ΣBPs	
Water	2015	Shenzhen, China, drinking water	81.0 ng/L Unheated 85.0 ng/L Heated	ND	ND	ND	ND	ND	ND	ND	[1]
	2016	TaiHu, China	17.5ng/L	ND	ND	ND	ND	ND	ND	ND	[2]
	2017	Beijiang, China	360 ng/L	ND	ND	ND	ND	ND	ND	ND	[3]
	2021 Summer	Jabalpur, India	0.884~1.42 μg/L	ND	ND	ND	ND	ND	ND	ND	[4]
	2021 Winter	Jabalpur, India	0.307~0.726 μg/L	ND	ND	ND	ND	ND	ND	ND	[4]
	2020	Bailong River, China, Wet Season	6.15~35.42 ng/L	n.d.~1.25 ng/L	n.d.~2.38 ng/L	ND	ND	ND	ND	ND	[5]
	2020	Jialing Rriver, China, Wet season	13.28~54.43ng/L	n.d.~2.25 ng/L	ND	ND	ND	ND	ND	ND	[5]
	2020	Nanhe River, China, Wet season	10.15~112.92ng/L	0.25~4.64 ng/L	n.d.~2.32 ng/L	ND	ND	n.d.~0.90 ng/L	ND	ND	[5]
	2020	Bailong River, China, Dry Season	11.60~59.28 ng/L	n.d.~0.58 ng/L	ND	ND	ND	ND	ND	ND	[5]
	2020	Jialing River,	18.90~80.95 ng/L	n.d.~0.76 ng/L	ND	ND	ND	ND	ND	ND	[5]

	China Dry Season									
2020	Nanhe River, China, Dry Season	12.45~153.62 ng/L	n.d.~4.99 ng/L	ND	ND	ND	ND	ND	ND	[5]
2021	Bailong River, China, Wet Season	12.51~186.76 ng/L	n.d.~1.78 ng/L	ND	ND	n.d.~0.9 ng/L	n.d.~17.9 ng/L	n.d.~2.77 ng/L	ND	[5]
2021	Jialing Rriver, China, Wet season	12.14~168.57 ng/L	n.d.~1.45 ng/L	ND	n.d.~5.7 ng/L	n.d.~1.36 ng/L	n.d.~3.3 ng/L	n.d.~4.6 ng/L	ND	[5]
2021	Nanhe River, China, Wet season	16.15~308.56 ng/L	0.32~57.3 ng/L	ND	n.d.~6.28 ng/L	n.d.~0.64 ng/L	n.d.~30.6 ng/L	n.d.~15.1 ng/L	ND	[5]
2021	Bailong River, China, Dry Season	15.2~98.22 ng/L	n.d.~0.8 ng/L	ND	ND	ND	n.d.~2.4 ng/L	n.d.~1.84 ng/L	ND	[5]
2021	Jialing River, China Dry Season	24.05~152.23 ng/L	n.d.~4.75 ng/L	n.d.~2.84 ng/L	ND	ND	n.d.~2.0 ng/L	n.d.~0.68 ng/L	ND	[5]
2021	Nanhe River, China, Dry Season	43.8~212.62 ng/L	n.d.~16.86 ng/L	n.d.~3.81 ng/L	ND	ND	n.d.~1.7 ng/L	n.d.~2.78 ng/L	ND	[5]

	-	Longyear byen City Landfill Leachate	ND	ND	ND	ND	ND	ND	ND	39 ng/L	[6]
	-	Longyear byen City River	ND	ND	ND	ND	ND	ND	ND	0.28 ng/L	[6]
Soil	-	Zhejiang, China	0.3 ± 0.1 ng/g	0.3±0.1 ng/g	0.8±0.2 ng/g	0.2±0.1 ng/g	n.d.	n.d.	1.6±0.2 ng/g	0.4~382.5 ng/g	[7]
	-	Mexico	<LOD~9.8 μg/kg	ND	ND	ND	ND	ND	ND	ND	[8]
	2017	Zunyi, China	377.5±228 μg/kg	ND	ND	ND	ND	ND	ND	ND	[9]
	2017	Shenzhen, China	29~6783 ng/g	3.8~333 ng/g	1.48~4913 ng/g	n.d~225 ng/g	n.d.~26.9 ng/g	n.d.~331 ng/g	<5 ng/g	186~11776 ng/g	[10]
	2016	Taihu, mud bottom	13.3 ng/g	ND	ND	ND	ND	ND	ND	ND	[2]
	2019	China	n.d.~692.0 ng/g	n.d.~0.868 ng/g	ND	ND	ND	ND	n.d.~43.828 ng/g	0.39~713.0 ng/g	[11]
	-	Longyear byen City Landfill	ND	ND	ND	ND	ND	ND	ND	3.7 ng/g	[6]
	-	Longyear byen City Soil	ND	ND	ND	ND	ND	ND	ND	3.0 ng/g	[6]
	-	Longyear byen City Sea floor mud	ND	ND	ND	ND	ND	ND	ND	0.18 ng/g	[6]
Atmosphere	2016	Shanghai, China	0.51 μg/g	ND	ND	ND	ND	ND	ND	ND	[12]
	2016	Shanghai, China, Office	0.35~2.67 μg/g	ND	ND	ND	ND	ND	ND	ND	[12]

2016	Shanghai, China, Supermarket	0.48~2.77 μg/g	ND	ND	ND	ND	ND	ND	ND	[12]
2016	Shanghai, China, Dormitory	0.20~4.70 μg/g	ND	ND	ND	ND	ND	ND	ND	[12]
2016	Shanghai, China, Residence	0.20~4.38 μg/g	ND	ND	ND	ND	ND	ND	ND	[12]
2011, Summer	Paris	0.076 ng/m^3	ND	ND	ND	ND	ND	ND	ND	[13]
2011, Winter	Paris	0.071 ng/m^3	ND	ND	ND	ND	ND	ND	ND	[13]
2011 夏	Loñez	0.084 ng/m^3	ND	ND	ND	ND	ND	ND	ND	[13]
2011 冬	Loñez	<LOQ	ND	ND	ND	ND	ND	ND	ND	[13]
2007	Chennai, India	4.55 ng/m^3	ND	ND	ND	ND	ND	ND	ND	[14]
2008	Mumbai, India	2.48 ng/m^3	ND	ND	ND	ND	ND	ND	ND	[14]
2007	Beijing, China	0.63 ng/m^3	ND	ND	ND	ND	ND	ND	ND	[14]
2007	Guangzhou, China	0.48 ng/m^3	ND	ND	ND	ND	ND	ND	ND	[14]
2009	Sapporo, Japan	0.34 ng/m^3	ND	ND	ND	ND	ND	ND	ND	[14]

“ND”：Indicates that no testing was performed

“n.d.”：Indicates no concentration detected

Reference

1. Yan, Y.; Huang, Q.; Mou, J.; Luo, W. Bisphenol A secondary pollution in barreled drinking water and its exposure assessment. *Practical Preventive Medicine* **2017**, *24*, 43–45.
2. Chen, M.; Guo, M.; Liu, D.; Li, J.; Zhang, S.; Shi, L. Occurrence and Distribution of Typical Endocrine Disruptors in Surface Water and Sediments from Taihu Lake and Its Tributaries. *Chinese Environmental Science* **2017**, *37*, 4323–4332.
3. Xiong, S.; Wang, X.; Luo, W.; Ma, Y.; Lin, Y.; Wang, M.; Zheng, J. Spatia Distribution, Ecological Risk and Industry-Dependence of Endocrine Disrupting Chemicals in the Beijiang River, South China. *Environmental Chemistry* **2021**, *40*, 3803–3814.
4. Kumawat, M.; Sharma, P.; Pal, N.; James, M.M.; Verma, V.; Tiwari, R.R.; Shubham, S.; Sarma, D.K.; Kumar, M. Occurrence and Seasonal Disparity of Emerging Endocrine Disrupting Chemicals in a Drinking Water Supply System and Associated Health Risk. *Sci Rep* **2022**, *12*, 9252, doi:10.1038/s41598-022-13489-3.
5. Zhao, B.; Tan, X.; Xue, M.; Lu, J.; Xu, D.; Yang, R.; Zhang, L.; Gou, W. Pollution Status and Distribution Characteristics of Bisphenols in Rivers of Guangyuan City. *Environmental Monitoring and Forewarning* **2023**, *15*, 17–23.
6. Wang, L. The Pollution characteristics and bioaccumulation of BPs in multiple media at Longyearbyen, Arctic. Master, Harbin Institute of Technology, 2022.
7. Xu, Y.; Hu, A.; Li, Y.; He, Y.; Xu, J.; Lu, Z. Determination and Occurrence of Bisphenol A and Thirteen Structural Analogs in Soil. *Chemosphere* **2021**, *277*, 130232, doi:10.1016/j.chemosphere.2021.130232.
8. Gibson, R.; Durán-Álvarez, J.C.; Estrada, K.L.; Chávez, A.; Jiménez Cisneros, B. Accumulation and Leaching Potential of Some Pharmaceuticals and Potential Endocrine Disruptors in Soils Irrigated with Wastewater in the Tula Valley, Mexico. *Chemosphere* **2010**, *81*, 1437–1445, doi:10.1016/j.chemosphere.2010.09.006.
9. Liu, C.; Yang, Y.; Yu, J.; Xu, J. Investigation and environmental risk assessment of octylphenol, nonylphenol and bisphenol A in the sediment of Xiangjiang River in Zunyi City.; Chinese Society for Environmental Sciences, 2019; p. 5.
10. Ye, Z. Studies on the Pollution Distribution of Typical Bisphenol Compounds in Soil and Earthworms and Toxicity Effects. Master, Shenzhen University, 2021.
11. Zhang, Y. Research on pollution characteristics and health risks of bisphenols in soil at national scale. Master, Harbin Institute of Technology, 2022.
12. Liu, W.; Wang, Y.; Liu, Y.; Sun, Y.; Wu, M.; Ma, J. Comparative assessment of human exposure to phthalate esters and bisphenol A from different indoor dust. *Journal of Shanghai University (Natural Science)* **2019**, *25*, 282–292.
13. Teil, M.-J.; Moreau-Guigon, E.; Blanchard, M.; Alliot, F.; Gasperi, J.; Cladière, M.; Mandin, C.; Moukhtar, S.; Chevreuil, M. Endocrine Disrupting Compounds in Gaseous and Particulate Outdoor Air Phases According to Environmental Factors. *Chemosphere* **2016**, *146*, 94–104, doi:10.1016/j.chemosphere.2015.12.015.
14. Fu, P.; Kawamura, K. Ubiquity of Bisphenol A in the Atmosphere. *Environmental Pollution* **2010**, *158*, 3138–3143, doi:10.1016/j.envpol.2010.06.040.
